# Editorial Notes

**Published:** 1909-12

**Authors:** 


### Editorial Notes.

**University of Bristol Matriculation.**

THE inaugural matriculation of a new University is perhaps of interest mainly to the acting staff and the students, who then initiate their University life. That this, the youngest University of the British Isles, has made a good start is shown by the 262 first matriculates, and the occasion was made impressive partly by the strong gathering of the professors and lecturers supporting the Vice-Chancellor, and by the goodly crowd of undergraduates.

**Christian Science and Faith Healing.**

THE *British Medical Journal* (October 23rd), commenting on Mr. Stephen Paget's attack on the supporters of Christian Science and Spiritual Healing at the recent Church Congress, remarks that "it is on false miracles that Christian Science has founded and built her church. She does not publish her failures." Mr. Paget insisted that in London the Society of Emmanuel was under the shadow of Christian Science. It has published the case of a man who was healed instantaneously, "in the last stage, apparently, of consumption in the lower part of the body"—a disease as mythical as Mrs. Eddy's malignant tubercular diphtheria—and the case of a man who was healed by laying-on of hands and annointing, "after one lung had sloughed away, and the other had half gone," and he is now "in perfect health, with two good sound lungs." One or two of these reported miracles have been investigated, with the result which might be expected. "Nothing," Mr. Paget added, "had happened yet in spiritual healing which had not its counterpart in mesmerism, treatment by suggestion, or commonplace medical attendance." Naturally the official defenders of Christian Science are up in arms; but that is a proof that they are hard hit. Dr. Johnson thought one of his pamphlets had been a failure because he had not been sufficiently attacked about it. Judged by this test, Mr. Paget has every reason to congratulate himself on the success of his plain speaking.

Mr. Stephen Paget further continued his attack in an article entitled "Where Christian Science Fails" (*Daily Mail*, October 15th, 1909), and also at the meeting of the Congregational Union at Sheffield, when he concluded his strong indictment of Mrs. Eddy and her ways as follows :—

"But her true strength, her ultimate secret, was neither in her sham philosophy nor in her devotions, but in her promise to heal all manner of diseases. She had healed and was healing many invalids. She helped them to forget or overcome those ailments which were called neurotic or neurasthenic. But she did not stop there. She loved to meddle with grave and urgent organic diseases, cancers, tumours, abscesses, hemorrhages, diphtheria, typhoid fever, acute inflammations, and destructive degenerations of the brain and spinal cord. Her advertised cures in such cases have been examined again and again, and have been found 'pitifully without foundation.' By patient inquiry amongst doctors and other friends, he had found the appalling and monstrous list of some of her victims.

"She has killed thousands—not by any mistake in diagnosis, for she never examines a case ; not by any mistake in treatment, for she does nothing that you can call treatment. Her feet are red with the blood of those whom she kills by sheer deliberate ignorance, by wilful blindness, by deliberate refusal to raise a finger for them. She puts them to death, and then, when they are dead, she says that they died of want of faith."

In a final impressive passage Mr. Paget said : "Her Christianity is all crown and no cross, all heaven and no earth, all comfort and no endurance, all pleasure and no pain. You turn to her works, and you find nothing in them that is really new, save the long trail of misery and preventable death. She tries in vain to hide it from our eyes. It proclaims her as 'one more wrong done to man, one more insult to God.'"

Dr. Horton's plea for toleration will receive little sympathy from the scientific and medical mind. Mr. Paget has done good service in the cause of scientific truth in opposition to the impostor and the quack, whether Christian or otherwise.

**The Control of Quackery.**

NOT only have the aberrations of the Eddy school been much in evidence of late, but also those of the Bodie school have been shown up in their audacious vagaries. The publication by the *British Medical Journal* of their booklet on *Secret Remedies, what they cost and what they contain,* has thrown a flood of light on the value of the advertisement columns of so many of our journals, many of which have declined to notice this book lest it might tend to the falling away of their revenue. We have no wish to depreciate the value of many proprietary compounds prepared with skill and care by competent pharmacists, and of which the composition is duly announced ; these are very commonly in use by medical men, but the secret remedies have no rational existence, and should not be encouraged by our journals because of the revenue they bring to the promoter and the advertiser.

We have now received another book, just published by Baillière, Tindall & Cox, which is mostly a reprint from the *Medical Press and Circular.* It is entitled *Quacks, False Remedies and the Public Health,* by Dr. David Walsh, who for many years has had the evils of irregular medical practice and of patent medicines constantly under his notice, and who now pleads for the long-expected Royal Commission, for which the need is extreme, as it is hopeless to expect that the lay newspapers will inform the public of the evils of a traffic which spends huge sums in advertisements. Much could be done by calling into play the powers of existing legislation, and the General Medical Council has already shown its desire, so far as its funds will allow, to check the modes of the irregular practitioner. But whilst the Government continues to receive a few hundred thousand pounds annually from stamps attached to secret nostrums which have been proved to be fraudulent, and so gives these frauds a Government stamp as of acknowledged and recognised authority and utility, the medical journals may continue to preach in vain. We warmly commend Dr. Walsh's book to all who have power to act on this important question.

**Health Committee, Bristol.**

At the meeting on November 17th, on the election of Chairman, the following remarks from the speech of Mr. Henry Anstey are worthy of note: "The low death-rate of Bristol was most gratifying. Smallpox had visited them during the year, and there were fears of a serious epidemic, but the intrusion was successfully resisted. Diphtheria, scarlet fever and other complaints caused them continual thought and occasional anxiety, but serious epidemics had been prevented, and cholera and plague had been kept from the port and city. This agreeable state of affairs was not merely the result of good luck; it was due to the labours and the precautions of the Health Committee under the guidance of Dr. Colston Wintle, assisted by a medical officer, Dr. Davies, of whom the whole city had cause to be proud, and a loyal and capable staff."

The Chairman, Dr. Colston Wintle, made special reference to the outlook as regards the treatment of consumption. He said, in conclusion, that there was another important matter to which he wished to allude, and he left it till the last for that reason—it was how they should take care and deal with all those suffering from consumption in their midst, and there were one or two points which he would like to place before them in dealing with the subject. At present they took in at Winsley only the most suitable cases, and that meant that the people who were very far gone they did nothing for whatever. They were a preventive committee, and it was their policy to prevent disease, but the very people who spread the disease were the ones they could do nothing for. It was important that they should do something for them. They sent cases to Winsley when they were in the early stages of consumption, but it was in the later stages of the disease that it became most infectious. More than two-thirds of those who applied to them had to be sent away, because they had no accommodation for them. What they wanted was to get colonies to which they could send those who came out of Winsley and look after them; also colonies for those who could not be taken to that institution owing to the lack of room or suitable condition. By this means only would that disease be at last stamped out. He spoke of the toll which the disease demanded every day, and said he was sure he would not appeal in vain to the members of that committee to grapple with this question, with the help of the Lord Mayor and citizens of Bristol.

**International Medical Congress, 1909. Budapest.**

As the next meeting of the great International Medical Congress will be held four years hence in London, it behoves us in this country to gather any lessons we may learn from those who attended at Budapest; hence the following note from the Professor of Anatomy of our University will be of interest, and also of some utility to the Committee of Organisation in this country. He writes :—

"The more one thinks of the Congress, the more one appreciates the enormous difficulties in the matter of management which must have faced the Committee of Management, difficulties which must be obvious to everyone. The mere fact that some five to six thousand medical men, women, and the wives—even the children in some cases—had to be accommodated would be enough to strike with dismay any committee, more especially as almost every nationality was represented, and some in great numbers. With the exception of the first day, on which, from the railway station to the Central Institute, something like pandemonium prevailed, everything, so far as our own personal experiences are concerned, went without a hitch.

"The Hungarians are charming hosts, and spared no effort to make their guests enjoy themselves.

"The magnificent hospitality, lavish to extravagance, must have created a lasting impression on all, and it was a matter of regret that this country was so poorly represented. Whether this was due to the fact that so few English people ever trouble to speak any language but our own, or even if they do are ashamed to use it, we know not, but the fact remains that we were there in very sparse numbers, and from remarks dropped, our kindly and Anglophile hosts felt it. The condition of the International room was a disgrace to our country. In one corner, the right-hand far corner, was a table devoted to Great Britain and Ireland. This table, apparently for the reception of current medical journals and lay newspapers, had on it a very poor portrait of His Majesty the King, a couple of out-of-date copies of the *Lancet* and *British Medical Journal*, and a few sheets of foolscap for the signatures of British congressists. That was its condition during the Congress. A glance at other tables showed them to be well stocked with medical literature and newspapers. It is to be hoped that this will be remedied in future congresses abroad.

"As we have before said, lavish hospitality was bestowed on all who cared to avail themselves of it; but, with their natural modesty, our hosts scarcely advertised the fact enough; for on going to what was modestly termed a reception, which in this country is usually interpreted as something which must be endured, with the aid of a little—sometimes very little—'stand-up' light refreshment, to which one may go at any time during the advertised hours, one found a really sumptuous dinner, which was not always easy to eat if one had already dined, as was the case until one realised what 'reception' really meant. At all these receptions it did one good to realise the good feeling which prevailed between the various members of Congress. In our own section, the anatomical, the utmost good feeling prevailed, and the arrangements were as nearly perfect as human imperfection can guarantee. The section had the advantages of not being overcrowded, and of being held in the luxurious Anatomical Institute of the University. Here were congregated such well-known exponents of the subject as Waldeyer, Apathy, Gaupp, Hammar, Laguesse, Maximow, Stieda, Sobotta, Osawa, Huntington, Lenhossék, Zellyesniczky, and many others, and the programme was sufficiently varied to interest all. The wonderful Institute in which the meeting was held makes one sigh when one turns to one's own country. Here are taught some five hundred students of anatomy, and although they are crying out for more 'subjects,' there is an annual supply of over one hundred and fifty.

"The city of Budapest well lends itself to such a Congress.

It is doubtful if there be any more beautiful city in the world. Its streets, wide as our 'Old Market,' with their rows of trees, their tramways—which run, by the way, near the footpath on each side, leaving a wide fairway to the middle—and the magnificent façades of houses by the side, unsullied by smoke, make striking pictures; and that majestic river, crossed by handsome bridges, five in all, which sweeps round flat Pest, separating it from hilly Buda, is a sight never to be forgotten, either when seen in the full blaze of sunlight, or by night with the full moon above, and the innumerable lights which illuminate not only its banks but its bridges below. To our mind the Congress was an unqualified success. Our hosts have set a high standard, which will be difficult even to reach when the Congress next meets, as it does, in London."

